# Supplementary material for: Novel Driver Strength Index highlights important cancer genes in TCGA PanCanAtlas patients
Source: PeerJ. 2022 Aug 11;10:e13860. doi: 10.7717/peerj.13860 (PMC9375969; doi:10.7717/peerj.13860)
Supplement: Supplemental Information 2 [file peerj-10-13860-s002.zip › COHORTS/genes plots/stage/2021_8_16_14_9_distribution_stage_males_II.pdf]

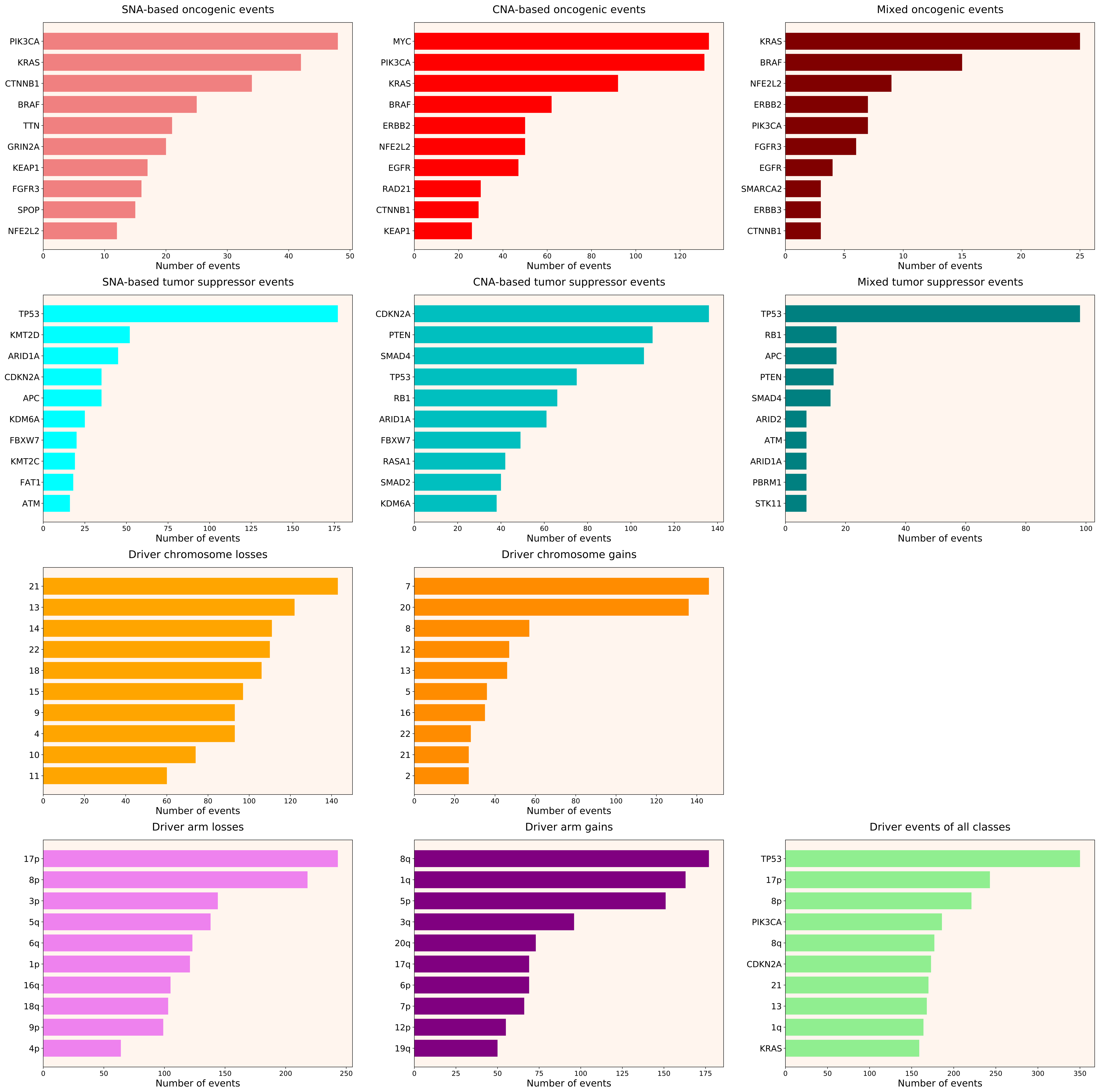
SNA-based oncogenic events
PIK3CA
KRAS
CTNNB1
BRAF
TTN
GRIN2A
KEAP1
FGFR3
SPOP
NFE2L2
0 10 20 30 40 50
Number of events
CNA-based oncogenic events
MYC
PIK3CA
KRAS
BRAF
ERBB2
NFE2L2
EGFR
RAD21
CTNNB1
KEAP1
0 20 40 60 80 100 120
Number of events
Mixed oncogenic events
KRAS
BRAF
NFE2L2
ERBB2
PIK3CA
FGFR3
EGFR
SMARCA2
ERBB3
CTNNB1
0 5 10 15 20 25
Number of events
SNA-based tumor suppressor events
TP53
KMT2D
ARID1A
CDKN2A
APC
KDM6A
FBXW7
KMT2C
FAT1
ATM
0 25 50 75 100 125 150 175
Number of events
CNA-based tumor suppressor events
CDKN2A
PTEN
SMAD4
TP53
RB1
ARID1A
FBXW7
RASA1
SMAD2
KDM6A
0 20 40 60 80 100 120 140
Number of events
Mixed tumor suppressor events
TP53
RB1
APC
PTEN
SMAD4
ARID2
ATM
ARID1A
PBRM1
STK11
0 20 40 60 80 100
Number of events
Driver chromosome losses
21
13
14
22
18
15
9
4
10
11
0 20 40 60 80 100 120 140
Number of events
Driver chromosome gains
7
20
8
12
13
5
16
22
21
2
0 20 40 60 80 100 120 140
Number of events
Driver arm losses
17p
8p
3p
5q
6q
1p
16q
18q
9p
4p
0 50 100 150 200 250
Number of events
Driver arm gains
8q
1q
5p
3q
20q
17q
6p
7p
12p
19q
0 25 50 75 100 125 150 175
Number of events
Driver events of all classes
TP53
17p
8p
PIK3CA
8q
CDKN2A
21
13
1q
KRAS
0 50 100 150 200 250 300 350
Number of events
